# The mouse "xenotropic" gammaretroviruses and their XPR1 receptor

**DOI:** 10.1186/1742-4690-7-101

**Published:** 2010-11-30

**Authors:** Christine A Kozak

**Affiliations:** 1 Laboratory of Molecular Microbiology, National Institute of Allergy and Infectious Diseases, Bethesda, MD 20892-0460, USA

## Abstract

The xenotropic/polytropic subgroup of mouse leukemia viruses (MLVs) all rely on the XPR1 receptor for entry, but these viruses vary in tropism, distribution among wild and laboratory mice, pathogenicity, strategies used for transmission, and sensitivity to host restriction factors. Most, but not all, isolates have typical xenotropic or polytropic host range, and these two MLV tropism types have now been detected in humans as viral sequences or as infectious virus, termed XMRV, or xenotropic murine leukemia virus-related virus. The mouse xenotropic MLVs (X-MLVs) were originally defined by their inability to infect cells of their natural mouse hosts. It is now clear, however, that X-MLVs actually have the broadest host range of the MLVs. Nearly all nonrodent mammals are susceptible to X-MLVs, and all species of wild mice and several common strains of laboratory mice are X-MLV susceptible. The polytropic MLVs, named for their apparent broad host range, show a more limited host range than the X-MLVs in that they fail to infect cells of many mouse species as well as many nonrodent mammals. The co-evolution of these viruses with their receptor and other host factors that affect their replication has produced a heterogeneous group of viruses capable of inducing various diseases, as well as endogenized viral genomes, some of which have been domesticated by their hosts to serve in antiviral defense.

## Introduction

Gammaretroviruses of three distinct host range tropisms have been isolated from the laboratory mouse (Table 1). The first of these mouse leukemia viruses (MLVs) were discovered in 1951 through their association with neoplasias of hematopoietic origin [1]. These MLVs were found to infect mouse and rat cells and could induce leukemias or lymphomas in inoculated mice. A second MLV type with a distinctly different host range was subsequently isolated by Levy and Pincus from the NZB mouse strain [2]. These viruses were defined by their apparent inability to infect cells of their host species, although they could efficiently infect cells of other species such as human, rabbit and cat [3,4]. These viruses were termed xenotropic (Gr. Xenos - foreign) to distinguish them from the mouse-tropic, sometimes pathogenic MLVs, now termed ecotropic (Gr. Oikos, home), that is, viruses with host range limited to the species of origin [5,6]. The third MLV host range group, the polytropic or dualtropic viruses (P-MLVs), are routinely isolated from mouse lymphomas and leukemias, and were initially described as having the broadest host range of the 3 MLV types because they could efficiently infect mouse cells as well as cells of heterologous species [7,8]. The P-MLVs can be pathogenic in mice and cytopathic in mink cells and have also been termed mink cell focus-forming (MCF) MLVs.

**Table 1:** Classically defined host range subgroups of infectious mouse gammaretroviruses isolated from laboratory mice.

			Host Range				
						
Type	Subtype	Tropism	Laboratory Mouse	Other Mammals	Receptor	Pathogenicity in Laboratory Mice	Endogenous Retroviruses
E-MLV		ecotropic	+	-	mCAT-1	+,-	*Emv*

X/P-MLV	X-MLV	xenotropic	-	+	XPR1	-	*Xmv*
	
	P-MLV	polytropic	+	+	XPR1	+,-	*M/Pmv *(*Pmv*, *Mpmv*)

The host range of these 3 MLV subtypes maps to the receptor binding domains (RBDs) of their envelope (Env) glycoproteins, and their RBDs govern the ability of these viruses to interact with their cognate receptors [9-11]. While the E-MLVs use the mCAT-1 receptor for entry [12], the X-MLVs and P-MLVs both use the XPR1 receptor [13-15] (Table 1) and I will term the set of mouse viruses that use this receptor, X/P-MLVs. Host range differences among the X/P-MLVs are due to sequence polymorphisms in the viral *env *and in the host receptor gene. These genes evolved in concert, altering virus-receptor interactions and the biological properties of these viruses, and producing an unusually heterogenous set of retrovirus and receptor variants.

Analysis of X/P-MLVs in laboratory and wild mice has detailed their roles in pathogenesis, their acquisition as endogenous elements in the *Mus *genome, and their interactions with and co-option as host restriction factors. This review will describe the evolutionary history of these viruses with special emphasis on tropism changes, the involvement of these viruses in disease induction in mice, and host factors that affect their replication and their recent transspecies transmission to humans.

## Endogenous MLVs in Laboratory Mice

Approximately 37% of the *Mus *genome is comprised of retroelements, and one-third of these are endogenous retroviruses (ERVs) [16,17]. ERVs represent germline proviral insertions generated by past retroviral infections. While the Class I ERVs that include the MLV ERVs constitute less than 1% of the mouse genome, attention has focused on this relatively small subgroup because of their relationship to the infectious and pathogenic C-type gammaretroviruses.

The MLVs and their endogenous ERV counterparts have the simplest of retrovirus genomes [18]. The MLV ERV genomes contain protein coding sequences for the virus core proteins (*gag*), enzymes (*pro*, *pol, in*) and envelope (*env*) that are flanked by long terminal repeat sequences (LTRs) that regulate transcription. The gammaretroviruses lack the accessory proteins of immunodeficiency viruses like HIV-1, have only one zinc-finger in nucleocapsid and rely on a translational strategy that reads through the *gag *termination codon. Many gammaretroviruses also uniquely produce a second, larger and glycosylated form of the Gag precursor polyprotein that uses an alternate, upstream initiation codon [19-21].

All three host-range MLV variants are present as ERVs in laboratory mice, many of which are full-length, with apparently nondefective protein-coding regions. Infectious viruses of all three host range classes can be isolated from mice, but not all ERVs produce virus, and those that do differ significantly in the timing and circumstances of their expression. Chromosomal locations for many of these ERVs in common inbred mouse strains were determined by conventional genetic methods [22-24] and the completion of the mouse genome sequence has allowed for complete characterization of the ERVs carried by the C57BL mouse [25]. ERV locations are, however, strain or strain-lineage specific; the various inbred strains carry multiple non-allelic provirus insertions [24,26].

### Ecotropic MLV ERVs *(Emvs)*

Many if not most of the *Emv*s can produce infectious virus. Up to 6 *Emv*s are found in the inbred strains (Table 2) [26]. Some of these *Emv*s are constitutively expressed from birth in the "high virus" strains such as AKR (Table 2) [27]. Other *Emv*s are inefficiently expressed, but their expression can be enhanced or induced by halogenated pyrimidines [28,29]; mouse strains carrying these *Emv*s produce infectious virus late in life, if at all (Table 2). Other mouse strains carry no *Emv*s [26]. Novel *Emv *proviruses can be acquired in viremic strains like AKR [30,31]; oocytes are the targets of these germline reinfections [32].

**Table 2:** Distribution of active MLV ERVs and their expression in selected common strains of laboratory mice.

ERV Type	Expression Level	Laboratory Mouse Strains^a^	Expressed MLV ERVs^b^
*Emv*	High	AKR, C58, HRS, PL, SL, F/St, C3H/Fg	2-6 *Emv*s/strain
	
	Intermediate	BALB/c, DBA, RF, CBA, NZW, C57BR, C57BL, C3H/He, SJL	1-2 *Emv*s/strain
	
	Low	NFS, NIH Swiss, C57L, 129, NZB, SWR	-

*Xmv*	High	NZB	*Nzv2, Nzv1*
		
		F/St	*Bxv1*
	
		C57BL, C57L, BALB/c, DBA, AKR, NZW, HRS	*Bxv1*
	Intermediate		
		MA/My	*Bxv1, Mxv1*
	
	Negative (Rare?)	NFS, NIH Swiss, A, 129, SWR	-

^a^[54,210].

^b^[26,55,57]

### Polytropic MLV ERVs *(M/Pmvs)*

There are up to 40 copies of P-MLV ERVs in the laboratory mouse genome [24,33]. The P-MLV ERVs have been divided into two closely related subgroups that differ most notably by the presence or absence of a 27-bp segment in the proline rich domain of *env*. These 2 P-MLV ERV groups are termed polytropic (*Pmv*s) and modified polytropic (*Mpmvs *or mPTs), and there is a smaller subgroup named intermediate polytropic MLVs (iPT), identified in NFS/N mice [34,35]. I will use the term *M/Pmvs *to refer to this subgroup of MLV ERVs collectively or when subtype is not known. Although the coding regions of many *M/Pmvs *have open reading frames [25], none are apparently capable of producing infectious virus; the reason for this is unknown, but may be due to accumulated mutations [25] or to LTR defects such as the presence of a 190 bp LTR insertion [36].

Despite the apparent inability of *M/Pmvs *to produce infectious virions, cell-to-cell transmission of this subgroup can be detected, and infectious P-MLVs can be produced in the presence of E-MLV infection. Thus, replicating E-MLVs can recombine with *M/Pmv *ERVs in mice to produce recombinant viruses with *M/Pmv env *sequences [35,37-40]; these viruses generally have polytropic host range, but are usually transmitted in viremic mice as pseudotypes of E-MLVs [41,42]. In the apparent absence of recombination, the transcribed products of *M/Pmvs *can also be packaged as homodimers into virions of exogenous ecotropic virus, and these "mobilized" *M/Pmvs *can infect cells, replicate in those new cells, and spread to other cells as pseudotyped virus [43]. Another transmission mechanism allows P-MLVs to completely bypass the need for their cognate receptor. These viruses are able to use alternative receptors in the presence of the soluble RBD glycoprotein for that receptor. Thus, entry defective E-MLVs as well as P-MLVs, but not X-MLVs, can be "transactivated" in this way by E-MLV RBD [44,45].

### Xenotropic MLV ERVs *(Xmvs)*

*Xmv*s are present in 1-20 copies per mouse strain [33,46]. The *Xmv*s in the sequenced C57BL genome are highly polymorphic, and phylogenetic analysis suggests that these *Xmv*s fall into 3 clades which may have resulted from 3 separate infections [25]. Some of the laboratory mouse *Xmv*s produce high levels of virus and other *Xmv*s can be induced to produce virus, but most *Xmv*s are not readily capable of producing infectious virus (Table 2). Among the laboratory mice, two strains, NZB and F/St, have a high virus phenotype, producing high titers of X-MLV throughout most of their lives [2,47,48]. Other strains rarely produce infectious virus, but cells from many common strains can produce virus following chemical induction or stimulation of spleen cells by bacterial lipopolysaccharide (LPS) or in a graft versus host reaction [28,29,49,50].

There are four active proviruses capable of producing virus in laboratory mice (Table 2). One of these proviruses, *Bxv1*, is a Chromosome 1 (Chr 1) locus sensitive to chemical induction or stimulation by LPS [51], and is carried by about one-third of the common strains of inbred mice [52]. The *Bxv1 *provirus has been identified [46,53], and is present in the sequenced C57BL genome [25]. Expression of *Bxv1 *is low except in the F/St strain, where the high level of virus expression is linked to the major histocompatibility locus [54]. The 3 additional active *Xmv*s found in laboratory mice have not been characterized. The high virus NZB mouse carries two loci neither of which maps to Chr 1 [55-57]. *Nzv1 *produces low levels of X-MLV, but *Nzv2 *is constitutively active [55]. The fourth active *Xmv *was identified in MA/My mice, a strain that also carries *Bxv1 *[57]. Other strains like NFS and SWR carry *Xmv*s but are rarely or not capable of producing infectious X-MLV [22,33,53] (Table 2).

### MLV ERV produced proteins

Many ERVs produce viral proteins in the absence of infectious virus. Some of these proteins were initially identified as novel antigens on mouse lymphocytes. Two of the most extensively studied of these antigens, G_IX _and XenCSA, are Env glycoprotein determinants [58,59]. These determinants can be detected in virus infected cells, and their expression in mice is associated with specific ERVs and is controlled by host genes linked to the major histocompatibility locus and the retrovirus restriction gene *Fv1 *[54,60,61].

### MLVs in cell lines and passaged tumors

The presence of multiple ERVs in the genomes of all laboratory mice can create problems for the use of these animals or established mouse cell lines in research. Many cell lines in common use carry active ERVs, or ERVs that can become active after long term culture of these lines. For example, the macrophage cell line RAW264.7 produces infectious E-MLV and P-MLV [62]. Also, various L cell derivatives like Clone 929 (ATCC CCL-1) and A9 (ATCC CCL-1.4) express Env glycoprotein and are either poorly infectible or completely resistant to infection by E-MLVs as well as P-MLVs (unpublished observations). Because *Xmv*s like *Bxv1 *can be induced by immune stimuli, including graft versus host reactions and B cell mitogens [49,50], it is not surprising to find infectious X-MLVs in hybridomas, or in tumor cells passed in SCID or *nude *immunodeficiency mice, as many of the strains carrying these mutations also carry *Bxv1*.

## Distribution of X/P-MLV ERVs in wild mouse species

**Figure 1:**
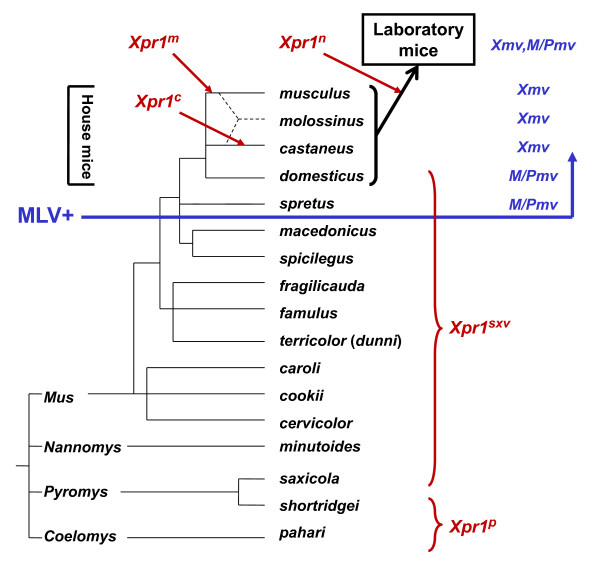
**Distribution of *Xpr1 *variants and endogenous X/P-MLV *env *genes in the genus *Mus***. The 4 subgenera originated about 7.5 million years ago (MYA). Red arrows and brackets mark the distribution of the 5 functionally defined *Xpr1 *alleles among *Mus *species and strains. The house mouse species are indicated by a bracket, and the specific MLV ERV *env *types found in *Mus *are listed on the right. The tree is adapted from the synthetic trees developed by Guenet and others [63,64,211].

**Figure 2:**
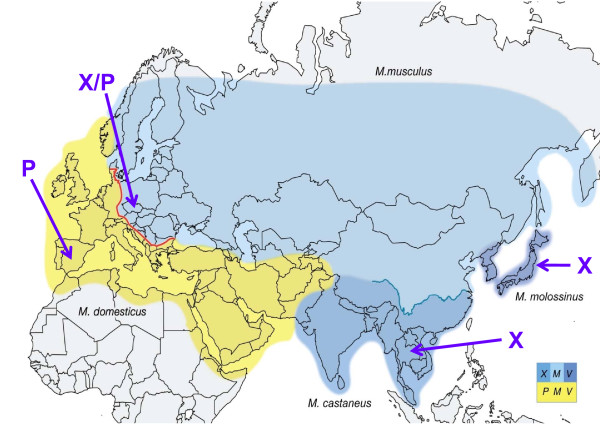
**Geographic distribution of the 4 house mouse species of *Mus *in Eurasia**. The three blue blocks show the distribution of species carrying primarily *Xmvs*, and the yellow block marks the range of the species carrying *M/Pmvs*. The blue line is the Yangtze River which roughly coincides with the transition between *M. castaneus *and *musculus *[66], and the red line represents the well-studied hybrid zone separating *musculus *and *domesticus *[211]. Infectious viruses of the indicated types were isolated from mice trapped at sites indicated with arrows; not shown: the X/P-MLV virus CasE#1 isolated from a California wild mouse.

The presence of still active MLV ERVs in mice and the positional polymorphism of these loci among inbred mouse strains indicate that all 3 ERV types entered the *Mus *germline recently. The genus *Mus *originated 8-12 million years ago (MYA) on the Indian subcontinent, and the 4 *Mus *subgenera diverged from one another shortly after *Mus *diverged from other *Murinae *[63,64] (Figure 1). Among the 40 recognized *Mus *species, there are 3 commensal species, or house mice, that evolved 0.5-1.0 MYA, and a fourth house mouse population in Japan, *M. molossinus*, which represents a natural hybrid of *M. castaneus *and *M. musculus *[65-67]. These house mice have largely nonoverlapping geographical ranges in Eurasia (Figure 2). House mice differ from their free-living or aboriginal ancestor species in their dependence on man; the house mice can live in houses, barns, warehouses and ships, and they travel wherever humans go [68]. Over the past few centuries, mice of the house mouse species were collected and interbred by hobbyists in Asia and Europe, and animals from these fancy mouse colonies were used to generate the common strains of the laboratory mouse [69,70]. It is also these house mouse species, the mice in closest contact with humans, that carry MLV ERVs.

**Figure 3:**
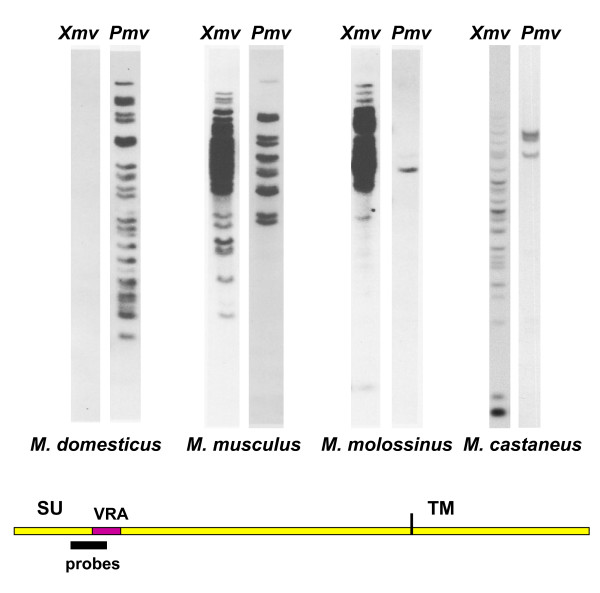
**Southern blot analysis of genomic DNAs of house mouse species using *env*-specific hybridization probes**. At the bottom is a diagram of the MLV *env *showing the position of the ~120 bp hybridization probes [33,71].

The identification of MLV ERV-related *env *and LTR sequences in house mouse species, but not their free-living progenitors, suggests these ERVs were acquired only 0.5-1.0 MYA [71]. Although inbred strains of laboratory mice tend to carry multiple copies of both *Xmv*s and *M/Pmvs*, these virus subtypes are largely segregated into different species in the house mouse complex [71] (Figure 1, 2 and 3). Sequences related to the *env *RBD of *M/Pmvs *are found in *M. domesticus *of Western Europe, while *Xmv*s predominate in *M. castaneus, M. musculus *and *M. molossinus *in eastern Europe and Asia (Figure 3). Use of probes from the LTR and from *env *segments that are outside the RBD largely confirmed this pattern of ERV segregation in *Mus *species, and found two polytropic subtypes, *Mpmvs *and *Pmvs*, in *M. domesticus *as well as evidence of atypical, recombinant types in the various house mouse species [72,73].

*Mus *is not native to the Americas, but was introduced with human travelers. American house mice most closely resemble the western European *M. domesticus *in that they lack *Emv*s and carry multiple *M/Pmv *ERVs and few or no *Xmv*s [71]. One exception to this is found in Lake Casitas, California, where mice carry multiple copies of *Xmv*s and *M/Pmvs *[71]. These mice also carry an *Emv *subtype common to Asian mice [71,74]. LC mice may thus represent a natural hybrid of European *M. domesticus *with *M. castaneus *mice that may have arrived in America with Chinese laborers and cargo.

Some of the wild mouse ERVs are active, and infectious viruses of xenotropic or atypical host ranges have been isolated from lymphoid tissues or cultured cells of Eurasian species and from mice trapped in California [57,75-79] (Figure 2). *M. molossinus *carries multiple ERVs capable of producing X-MLVs [57], one of which has been identified as the active laboratory mouse *Bxv1 Xmv *[52]. *Bxv1 *is found in some, but not all *M. molossinus *breeding lines, but has not been identified in the *Xmv*-positive progenitors of *M. molossinus*, *M. musculus *and *M. castaneus*. This indicates that the *Bxv1 *insertion arose relatively recently in the Japanese *M. molossinus *mice. The presence of Japanese mice among the fancy mouse progenitors of the laboratory strains [80,81] also suggests that these strains acquired *Bxv1 *from Japanese mice. Other wild mouse species, like *M. dunni *and *M. spretus*, carry only *M/Pmvs*, and these ERVs, like their laboratory mouse counterparts, do not produce infectious virus. However, *M. spretus *can, like laboratory mice, produce infectious P-MLVs when inoculated E-MLVs recombine with *M/Pmv *ERVs [82].

## Heterogeneity among Infectious X/P-MLVs

Many laboratory and wild mice carry ERVs that can produce infectious MLVs, and some wild mouse populations also carry infectious MLVs that have not become endogenized [83,84]. The various X/P-MLVs isolated from laboratory and wild mouse species differ phenotypically on the basis of host range, variable reactivity with anti-MLV antibodies, cross-interference, cytopathicity, and pathogenicity in mice. Sequence data for these viruses is limited, but comparisons of available *env *sequences indicate there is significant heterogeneity, particularly in the RDB of the Env glycoprotein. This region is marked by 3 hypervariable segments, VRA, VRB, VRC, where multiple substitutions and indels distinguish the prototypical P-MLVs and X-MLVs. In addition to these sequence polymorphisms, another source of variation comes from the fact that each infectious P-MLV is the product of a recombination between E-MLVs and different endogenous *M/Pmvs*, and the size of the recombination can vary [82,85,86].

Not all laboratory mouse P-MLVs have polytropic host range. Some of these recombinant viruses (R-XC^-^, SL3-2, GPA-V2, ecotropic recombinants) have ecotropic host range [9,87-89]. These tropisms are governed by RBD substitutions that lie outside the major host range determinant for MLVs, VRA, which is the most 5' of the 3 variable regions of the *env *RBD [9,11,90]

Among the wild mouse isolates, X-MLVs from *M. molossinus *and *M. castaneus*, and P-MLVs from *M. spretus *resemble the laboratory mouse isolates in their restriction maps and biological properties [78,91], but X/P-MLVs with atypical host range have also been isolated from wild mice. One such isolate, CasE#1 (or Cas E No. 1), was isolated from a wild-trapped California mouse [77]. It resembles P-MLVs in its ability to produce MCF-type foci and in its interference properties, but, like X-MLVs, it fails to infect laboratory mouse cells and has novel receptor requirements [77-79]. Cz524 MLV was isolated from the wild derived *M. musculus *strain CZECHII/EiJ, and differs from both P-MLVs and X-MLVs in host range [79]. The *env *genes of these two wild mouse isolates are not identical to laboratory mouse P-MLVs or X-MLVs, but are related to both [78,79].

## XPR1 Receptor for X/P-MLVs

The X-MLV and P-MLV subgroups use the same XPR1 receptor for entry, although they were initially described as 2 host range groups because of their differential ability to infect mouse cells. This receptor was first identified as a P-MLV susceptibility gene and was mapped to distal Chr 1 [92]. Subsequent studies showed that X-MLVs could infect cells derived from wild mice [93-95], and genetic crosses mapped this X-MLV susceptibility as well as the P-MLV resistance of *M. castaneus *to the same segment of distal Chr 1 [95,96]. The conclusion that susceptibility to X-MLVs and P-MLVs is governed by a single gene was also supported by the observations that these viruses cross-interfere [77,97], and that infection by X-MLVs in wild mice is reduced by *Rmcf*, a host gene that restricts P-MLV infection by receptor interference [95].

The XPR1 receptor for X-MLVs and P-MLVs has 8 putative transmembrane domains and 4 putative extracellular loops [13-15]. This multiple-membrane-spanning structure is a common feature of the receptors used by the gammaretrovirus family [98]. While this suggests these viruses evolved from a common progenitor, this multi-membrane spanning structure is not representative of all retroviral receptors, some of which, like the lentivirus CD4 receptor and the receptors for alpha- and betaretroviruses have single TM domains [99]. Although the host cell function of XPR1 has not been defined, the other gammaretrovirus receptors with known function have all been identified as transporters of small solutes like phosphate or amino acids [98]. The XPR1 protein may have a similar function as it is homologous to the yeast SYG1 and plant PHO1 genes, which have roles in signal transduction and phosphate sensing and transport, respectively [14]. Recent work has indicated that XPR1 is upregulated following activation of the NF-κB RANKL-RANK signaling pathway in osteoclastogenesis [100].

**Figure 4:**
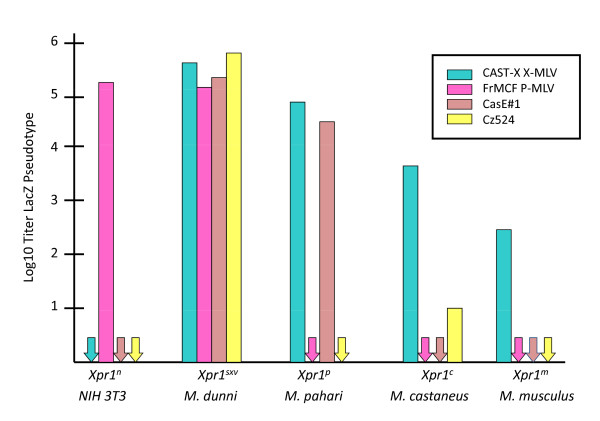
**Five functional variants of *Xpr1 *in *Mus***. Susceptibility to 4 host range X/P-MLV variants was determined using virus pseudotypes carrying the LacZ reporter gene [101].

*Mus *species and inbred strains carry at least 5 functionally distinct XPR1 variants [13-15,78,95,96,101]. These five *Mus *XPR1 s differ in their ability to support entry by prototype X-MLVs and P-MLVs and by the two wild mouse isolates CasE#1 and Cz524 (Figure 4) [79,101]. One of these alleles, *Xpr1^sxv ^*(susceptibility to xenotropic virus), is fully permissive for all X/P-MLVs. The other 4 variants restrict infection by two or more members of this virus family. All variants except the XPR1 of NIH 3T3 cells support entry by X-MLVs, although with differences in efficiency. Only 2 of the 5 receptor variants are permissive for P-MLVs. The laboratory mouse allele, *Xpr1^n^*, allows entry only by P-MLVs.

**Figure 5:**
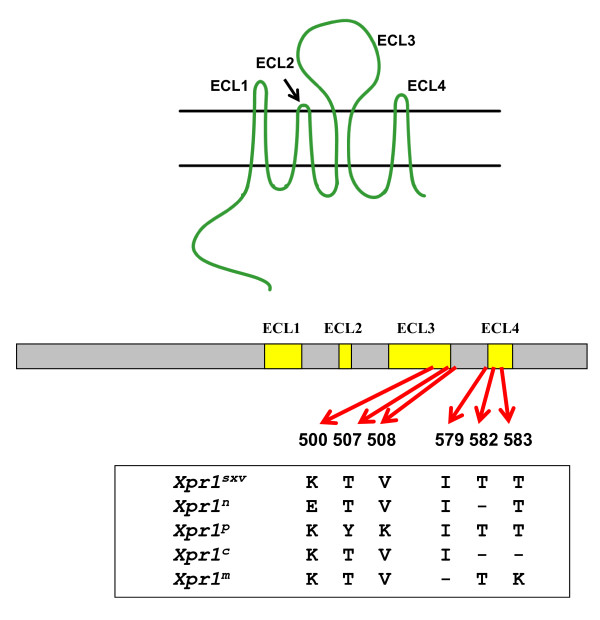
**Putative transmembrane structure of XPR1 and locations of the 6 residues responsible for receptor function**. XPR1 has 4 putative extracellular loops (top) indicated as yellow bars in the mRNA. Codon positions for residues involved in entry are marked with arrows, and residues at these sites are shown for the 5 *Mus *alleles. "-" represents a deletion.

Specific XPR1 residues responsible for virus entry lie in 2 of the 4 predicted extracellular loops (ECLs) of *Xpr1*, ECL3 and ECL4 (Figure 5) [78,79,101-103]. Two critical amino acids are needed for X-MLV entry, K500 in ECL3, and T582 in ECL4 [102]. Both sites are mutated in the X-MLV restrictive NIH 3T3 *Xpr1^n ^*allele, and corrections at either of these two sites produce X-MLV receptors [102], although these are not functionally equivalent. Thus, the Δ582T insertion generates a receptor for X-MLV as well as CasE#1, but the E500K substitution does not allow for CasE#1 entry [78]. Sensitivity to different X/P-MLVs is further modulated by specific substitutions at ECL3 residues 500, 507, 508 and ECL4 residues 579 and 583 [78,79,101] (Figure 5). Substitutions at these sites can result in subtle differences in the efficiency of virus infection or complete resistance to specific X/P-MLVs.

All of the viruses that use XPR1 are sensitive to mutational changes in both ECL3 and ELC4, suggesting that residues in these ECLs contribute to a single virus attachment site [78,79,101]. Thus, *Xpr1 *mutants with substitutions in ECL3 but identical ECL4 sequences produce receptors with differential sensitivities for P-MLVs and for the CasE#1 and Cz524 viruses. These same viruses also differ in their infectivity for cells with *Xpr1^m^*, *Xpr1^c ^*and *Xpr1^sxv^*, which have identical ECL3 sequences but different deletions in ECL4. The requirement for residues in two XPR1 loops for receptor function is not unusual as other receptors require multiple domains [104]. While these multiple domains in several other retroviral receptors have distinctive roles in virus attachment and entry [105,106], this has not been shown to be the case for the XPR1 ECL3 and ECL4 domains.

## Evolution of the *Xpr1 *receptor gene in virus infected mice

The 5 functionally distinct mouse XPR1 receptor variants are found in different mouse lineages. The species and geographic distribution of these variants indicate that much of this receptor variation is coincident with exposure to MLVs [101]. Most *Mus *species carry the most permissive XPR1 variant, *Xpr1^sxv^*, which persisted in *Mus *through much of its evolutionary history (Figure 1). The species with *Xpr1^sxv ^*either lack X/P-MLV ERVs or carry only M/PMV ERVs that are not known to produce infectious virus. The 4 restrictive receptor alleles appeared at two distinct time points in *Mus *evolution. *Xpr1^p ^*appeared about 7.5 MYA, shortly after the divergence of *Mus *from other *Murinae *[63,64], and there is no evidence that the mice with this restrictive receptor were exposed to MLVs as they lack MLV ERVs [71]. The other 3 restrictive *Xpr1*s arose later, in the house mouse complex. This roughly coincides with the acquisition of X/P-MLV ERVs (Figure 1). Two of these 3 restrictive house mouse variants, *Xpr1^m ^*and *Xpr1^c^*, like the presence of *Xmv *sequences in these species, show an apparent species-wide distribution [101], suggesting these variants provided a survival advantage.

**Figure 6:**
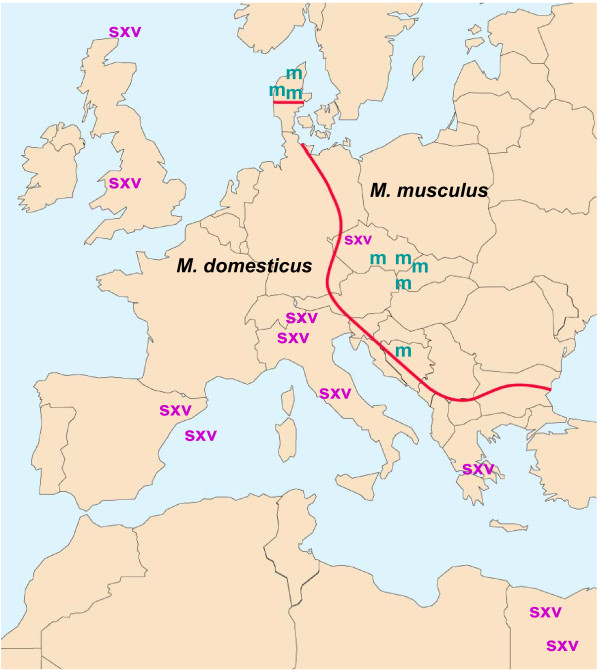
**Distribution of *Xpr1^sxv ^*and *Xpr1^m ^*in mice trapped in various sites in Europe**. The red line represents the 20 km-wide hybrid zone separating the ranges of *M. domesticus *and *M. musculus *[212]. Symbols indicate the trapping sites of each sequenced sample [101].

*Xpr1^n ^*is the only one of the 5 *Mus Xpr1 *alleles to completely restrict X-MLVs, and its species of origin is unclear. This laboratory mouse allele has not been found in any wild mouse [101]. The common inbred strains of the laboratory mouse represent genomic mosaics of the various house mouse species, but *M. domesticus *is the largest contributor (~92%) to the inbred mouse genome [69]. The expectation that *M. domesticus *would likely carry *Xpr1^n ^*also makes biological sense, as these mice carry endogenous *Pmv*s but not *Xmvs *consistent with *Xpr1^n ^*receptor function [71]. However, *M. domesticus *mice trapped at various sites throughout its western European range and in the Americas all carry *Xpr1^sxv ^*(Figure 6). It is thus possible that *Xpr1^n ^*arose later, in the fancy mouse progenitors of laboratory mice. These fancy mouse interspecific hybrids would have acquired *M/Pmvs *from *domesticus *and *Xmv*s from *musculus *and *castaneus*, and a restrictive receptor might have provided a survival advantage for these mice.

Sequence comparisons of *Xpr1 *orthologues from *Mus *and other rodent species indicate that there is substantial polymorphism in the short, virus-binding 13 residue ECL4. This region contains 3 residues that are conserved in all mammalian XPR1 orthologues, but these residues do not contribute to receptor function [101]. While ECL4 sequence variation is due largely to replacement mutations, the three restrictive alleles found in virus-infected house mice, *Xpr1^m^*, *Xpr1^n^*, *Xpr1^c^*, all carry deletions in this region (Figure 5) [101,102]. The deletions are all different and no deletions in this region are found in other mouse or rodent species, or in any mammalian *Xpr1 *orthologue. Either the 6 residues involved in these deletions are critical for entry as has been shown for some of them, or decreasing the size of the ECL4 loop may effectively disable receptor function.

## XPR1 variants in inbred strains of the laboratory mouse

The first *Xpr1 *allele to be recognized, *Xpr1^n^*, was identified in X-MLV resistant laboratory mice, but *Xpr1^n ^*is not universal among the common inbred strains of laboratory mice. These widely used common strains were developed largely by William Castle and C. C. Little from fancy mice provided by hobbyists, especially Abbie Lathrop [70]. While these Lathrop/Castle/Little strains have a shared ancestry reflected in their reduced genetic diversity compared to *Mus *species [107,108], the various lineages and strains differ in their susceptibility to virus induced disease, and in their ability to produce infectious MLVs or viral proteins (Table 2). While some of these differences can be explained by the presence of ERVs with different levels of activity, receptor variations could also be important factors in these different phenotypes.

While *Xpr1^n ^*is carried by the majority of laboratory mice, *Xpr1^sxv^*, which encodes the permissive receptor, has now been identified in several common inbred strains. Cells from these strains can be infected with X-MLV [52]. One of the strains carrying *Xpr1^sxv^*, F/St, is one of the two strains that produce high levels of X-MLVs throughout life (Table 2). The role of the receptor in this phenotype is unclear; however, as F/St viremia requires genes on Chr 17 near the major histocompatibility locus and in the segment of distal Chr 1 which carries the active *Xmv *provirus *Bxv1*, as well as *Xpr1 *[47,54].

Inbred strains derived from various wild mouse species are available that carry all 4 of the wild mouse *Xpr1 *variants as well as *Xpr1^n^*. These strains can, in principle, be used to determine if receptor-mediated secondary spread affects virus levels in mice carrying active proviruses like *Bxv1*. These mice can also be used to develop models to describe the time course, tissue tropism and pathogenic consequences of exogenous infection by the different X/P-MLV host range subtypes, and to determine whether receptor variants affect the type of recombinant viruses that appear.

## Transspecies transmission and XMRV

The X/P-MLVs are capable of infecting cells of other species, including humans. In fact, cells of nearly all mammals are permissive to infection by X-MLVs, and a smaller subset of these is also susceptible to P-MLVs [3,4,77,101] (Table 3). The horizontal transfer of infectious MLVs between individuals has been documented in wild mouse populations and in laboratory mice [109,110]. MLV-infected house mouse species have a worldwide geographic distribution [111], and are considered important vectors of diseases that infect humans and their livestock [112]. It is therefore not surprising that MLV-related ERVs are found in the genomes of amphibians, reptiles, birds and mammals [113], and that X/P-MLV-related viruses and viral sequences have now been reported in humans [114-119].

**Table 3:** Infectivity of X/P-MLVs and XMRV on cells of mammalian species

	Log_10 _Titer*				
	
Cells	CAST-X X-MLV	XMRV	Cz524	CasE#1	MoMCF P-MLV
*M. dunni*	+++	+++	+++	+++	+++

Human 293	+++	+++	+++	+++	+++

Monkey COS-1	+++	+++	+++	+++	+++

Ferret	+++	+++	+++	+++	+++

Rabbit SIRC	+++	++	+++	+++	++

Cat CRFK	+++	+++	+++	+++	+++

Bat Tb-1-Lu	+++	++	+++	+++	-

Guinea pig JH4	+++	++	++	-	-

Goat	+++	++	+	-	-

Buffalo	+++	+	-	-	-

Dog MDCK	+++	++	-	-	-

Gerbil GeLu	+++	-	-	-	-

Chinese hamster Lec8	+++	-	-	-	-

*Infectivity measured as the number of β-galactosidase-positive cells in 50 μl of viral pseudotypes carrying the LacZ reporter. Log_10 _titer: +++, >3; ++, 2-3; +, 1-2; -, 0-1. [101]. Hamster Lec8 cells have a glycosylation defect that relieves resistance to some X-MLVs.

Infectious virus related to X/P-MLVs has been isolated from human patients with prostate cancer and chronic fatigue syndrome [115,117,118]. This virus, termed XMRV (xenotropic murine leukemia virus-related virus), shows close sequence homology with X/P-MLVs [114], uses the XPR1 receptor [115], and has xenotropic host range [79]. The VP62 isolate of XMRV and the sequenced DG75 X-MLV genome [120] show overall 94% sequence identity [114]. A more complicated picture emerges from sequence comparisons of the XMRV coding and non-coding domains with corresponding regions of X-, P-, and E-MLVs, as well as the active *Bxv1 Xmv *and a full length *Mpmv*. While XMRV most closely resembles the X-MLVs in SU*env *and LTR, it shows greater identity to *M/Pmvs *in *gag *and *pol *(Table 4). This, coupled with the recent finding of *M/Pmv *related *env *and *glycogag *sequences in human blood donors and chronic fatigue patients [119] points out the need for further work to clarify the evolutionary path linking the human and mouse viruses and to describe the epidemiology of this virus family in wild mice [121].

**Table 4:** Sequence comparisons of coding and non-coding domains of XMRV and 5 full length gammaretrovirus genomes.

		DG75 X-MLV AF221065	*Bxv1 Xmv *AC115959	*Mpmv1 Pmv *AC127565	MCF1233 P-MLV U13766	AKV E-MLV J01998
**LTR**	**U5**	90	100	90	99	93
	
	**R**	95	100	96	99	95
	
	**U3**	87	94	85	84	84

***gag***	***gag *leader**	85	86	90	85	85
	
	**MA**	90	86	98	84	87
	
	**p12**	97	81	99	82	81
	
	**CA**	99	88	99	89	87
	
	**NC**	98	96	99	92	92

***pol***	**PR**	99	92	99	92	92
	
	**RT**	94	93	95	92	93
	
	**IN**	92	93	97	89	86

***env***	**SU**	94	95	89	90	< 75
	
	**TM**	98	98	98	83	81

Numbers represent percent identity. DG75 is an X-MLV isolated from the human DG-75 lymphoblastoid line [120], MCF1233 and AKV MLV are infectious viruses isolated from AKR strain mice. *Bxv1 *is the active endogenous xenotropic ERV found in strains such as C57BL and BALB/c. AKV has a duplicated enhancer in U3 that was not included in the analysis. *Mpmv1 *is a full-length ERV in the sequenced C57BL genome; it contains a 190 bp LTR insert that was not included in the analysis. GenBank accession numbers are provided for the 5 sequences; comparisons were done with VP62 XMRV NC_007815.

The XMRV virus and X/P-MLV sequences found in humans may have been acquired directly from mice, or after transmission from mice to another species in contact with humans. If there is direct transmission from infected mice, this could be reflected in the geographic distribution of virus and/or receptor type in mice and the worldwide incidence of prostate cancer. Studies have reported very different rates of XMRV detection in prostate cancer patients (reviewed in [122]), and while these differences may have technical explanations, it is also possible that some of these differences are due to geographic differences in exposure to XMRV. The highest rates of prostate cancer are found in the U.S. and lowest rates are found in Asian countries like Japan, India and China [123]. Rates in Europe are lowest in Eastern European countries. This distribution generally corresponds to the distribution of *Xpr1 *receptor variants in mouse populations; the most permissive allele, *Xpr1^sxv^*, is found in high tumor incidence areas, and the most restrictive allele, *Xpr1^m^*, is found in low tumor areas like Japan and eastern Europe. Mice in low tumor areas of Asia also carry receptor blocking genes [124] further indicating that these mice might be poor candidates for zoonotic transmission to humans. While these observations are suggestive of direct transmission between mice and man, it should also be noted that mice in areas of high tumor incidence are not known to carry infectious X/P-MLVs or expressed MLV ERVs.

The transmission of XMRV to humans was likely accompanied by adaptive changes, and the observed sequence and phenotypic differences of XMRV relative to the X/P-MLVs have focused particular attention on the *glycogag *leader region, LTR and *env*. XMRV carries unusual deletions in *glycogag*, a region that in E-MLV influences virus release and sensitivity to interferon [125] and also inhibits the activity of the host cell antiretroviral factor APOBEC3 [126]. XMRV differs from MLVs in its affinity for and efficient replication in prostate cells, and this has been attributed to the glucocorticoid response element in the XMRV LTR U3 [127-129]. Finally, XMRV has a novel host range and receptor requirements that distinquish it from the mouse X/P-MLVs. Thus, the XPR1 receptor determining residues K500 and T592 produce equivalent receptors for X-MLV but not for XMRV [101]. Also, while the mouse X-MLVs are generally able to infect all mammals, XMRV is uniquely restricted by Chinese hamster and gerbil cells (Table 3), a restriction associated with sequence differences in the receptor determining region of *Xpr1 *ECL4 [101]. These multiple XMRV differences may represent adaptations acquired through contact with humans or with an as yet undiscovered species before transmission to humans.

## Pathogenesis by MLVs

The detection of XMRV and P-MLVs in various human patient groups and in blood donors raises questions about the pathogenic and mutagenic potential of these viruses in humans and concerns about the safety of the blood supply. While the involvement of these viruses in human disease is still under investigation, the MLVs were recognized as disease-inducing agents in mice almost 60 years ago [1]. Although most MLVs are generally non-pathogenic or poorly pathogenic in mice, MLVs can and do cause disease in their natural hosts, and the induction of disease can involve X-MLVs and P-MLVs as well as E-MLVs.

Mouse strains carrying active *Emv*s, like AKR, HRS, and C58, have a high incidence of spontaneous lymphomas, and mice inoculated with specific MLVs can develop diseases such as lymphocytic leukemia, erythroleukemia, immunodeficiencies, and neurological diseases. The naturally occurring and induced neoplastic diseases are generally induced, following a long latency period, by insertional mutagenesis. In this process, novel virus integrations activate genes involved in growth regulation or inactivate tumor suppressor genes [130,131]. The established role of insertional mutagenesis in MLV-induced disease prompted the characterization of XMRV insertion sites in human prostate cancers [132]. While no common insertion sites were identified near recognized proto-oncogenes or tumor suppressor genes, XMRV integrations were found near cancer breakpoints, common fragile sites, microRNAs, and cancer-related genes.

In mice, MLV-induced neoplastic disease is often associated with the *de novo *generation of infectious and pathogenic P-MLVs. The disease process generally begins with the establishment of chronic infection with E-MLVs. These viruses can recombine with *M/Pmvs *and *Xmv*s to generate recombinant infectious virus with P-MLV host range and increased virulence [133,134]. These P-MLV recombinants can be cytopathic, which is why they were initially termed mink cell focus-forming viruses or MCF MLVs [8]. Although not all virus-induced diseases are accompanied by the generation of P-MLV recombinants, the importance of MCF MLVs in the disease process is supported by the fact that these recombinants are found in lymphoid tissues of preleukemic mice and can be found in tumors as infectious virus and novel integrations [135]. Also, inoculation of neonatal AKR mice with MCF virus accelerates the appearance of thymomas [136], and disease is restricted in mice carrying the *Rmcf *resistance gene that inhibits replication of P-MLV [137] or in mice inoculated with genetically altered viruses that cannot participate in MCF production [138].

The recombinations that generate infectious pathogenic P-MLVs involve at least two segments of the viral genome, *env *and LTR. The LTR sequences are contributed by the active *Xmv*, *Bxv1 *[53,139], and the LTRs of AKR mouse MCFs have duplicated enhancer regions not found in the endogenous *Bxv1 *proviral sequence [134]. The recombinant *env *segment in MCF MLVs can vary due to the sequence of the participating *M/Pmv *[35] as well as the size of the recombinant segment. Recombinational breakpoints in the MCF *env *tend to cluster in 2 segments of the 3' half of SU*env *or in the 5' end of TM*env *[82,85,86].

The role of the recombinant *env *genes in the disease process is incompletely defined, but these substitutions can contribute to the target cell specificity and disease type induced by MCF MLVs. The most well-studied example of disease mediated by viral Env is the rapid erythroleukemia induced by Friend SFFV, a replication-defective MCF-type recombinant. SFFV encodes a unique 52/55 kDa Env-related protein that functions as an oncogene and induces disease by activating signal transduction pathways associated with the erythropoetin receptor and the receptor tyrosine kinase Stk [140-142]. For other pathogenic MCF MLVs, Env may support the *in vivo *progression of tumors by hampering the immune response. In some cases, *Env *substitutions may facilitate virus evasion of the immune system [143], or the ERV-derived *env *genes expressed in tumors may contribute to a T-cell mediated subversion of immune surveillance that allows for tumor cell proliferation [144,145].

Preleukemic thymuses can contain large amounts of unintegrated MCF MLV DNA resulting from failure to establish superinfection interference [135,146]. Such superinfections have been associated with cytopathic killing by other pathogenic retroviruses such as HIV and ALV [147,148], and superinfection by MCF results in lymphocyte depletion in the thymus of infected mice [149]. This depletion may result from endoplasmic reticulum stress induced apoptosis [150]. The ability of MCF MLVs to evade superinfection interference is unusual since other MLVs effectively prevent multiple infections by receptor downregulation. This phenomenon may be explained by two properties of the MCF Env. First, like some other pathogenic retroviruses, MCFs may have lower receptor-binding affinity [45,102]. Second, multiple infections can result from the ability of MCFs to use the E-MLV receptor for entry in the presence of soluble E-MLV Env [45].

## Host factors that restrict replication of X/P-MLVs and XMRV

The acquisition of MLV ERVs, the time course and tissue specificity of their expression, and the transmission of these viruses to new hosts are governed by host factors that restrict or enhance virus replication and spread. These host factors include the innate and acquired immune systems, as well as numerous constitutively expressed antiviral factors that inhibit virus replication, many of which were initially identified in studies on the mouse gammaretroviruses. These factors can block or interfere with different stages in the viral life cycle, such as virus entry, uncoating and reverse transcription, integration, assembly and release. For this review, I will focus on the host factors that either specifically target the X/P-MLVs and XMRV, or factors that have been shown to have significant restrictive effects on these viruses (Table 5). Among the antiviral factors that restrict these gamaretroviruses, some, like APOBEC and tetherin/BST2 are broadly antiviral, whereas Fv1 targets only MLVs, while XPR1, LVIF, and the RMCF-like interference genes restrict only X/P-MLVs.

**Table 5:** Host restriction factors that inhibit replication of gammaretroviruses.

	Restriction*			
	
Restriction Factor	X-MLV	P-MLV	XMRV	E-MLV
* Xpr1*	+	+	+	-

Entry: glycosylation	+	-	-	+

* Lvif*	+	+	?	-

ERV interference	?	+ (*Rmcf *genes)	?	+(*Fv4*)

* Fv1*	+	+	+	+

* Apobec3*	-?	+?	+	+

* Tetherin*/BST2	?	?	+	+

*+, infectious virus known to be inhibited; determination of *Apobec3 *restriction of X/P-MLVs based on mutational patterns found in ERVs [25].

### *Xpr1* receptor polymorphism and glycosylation blocks to entry

Receptor polymorphisms can clearly provide an especially effective antiviral defense. As already noted, 4 of the 5 XPR1 receptor variants in *Mus *restrict two or more viruses in the X/P-MLV family. These restrictions result from deletion mutations or replacements that have been shown to display a pattern of positive selection suggesting an evolutionary history of genetic conflicts [101]. Furthermore, 3 of the 4 naturally occurring restrictive receptor alleles evolved in virus infected mice, suggesting that these variants provided a survival advantage. Additional restrictive *Xpr1 *variants are found in non-*Mus *mammals [101] (Table 3).

Entry can also be blocked by factors that interfere with receptor function. Glycosylation of cellular proteins is associated with resistance to E-MLVs in rodent cells and X-MLVs in Chinese hamster cells [151-153]. The glycosylation block in hamster cells does not affect all X/P-MLVs; inhibition of glycosylation relieves resistance to most X-MLVs, but not to P-MLVs or to XMRV [79]. Although the XPR1 protein contains multiple sites for N-linked glycosylation including several in the ECL3 receptor determining region, it is not clear whether the glycosylation entry block affects the receptor or another as yet unidentified glycoprotein.

### Leukemia virus inactivating factor (LVIF)

Mice produce a serum factor, leukemia virus interfering factor (LVIF), that inactivates X-MLVs [154]. This factor is stable when exposed to acid pH, ether, proteases and temperature extremes [155]. LVIF is separable from immunoglobulin, is found in the lipoprotein fraction of serum and is sensitive to antiserum to apolipoproteins [156,157]. This factor inactivates X-MLVs and P-MLVs but not E-MLVs or the wild mouse amphotropic MLVs. LVIF is therefore not equivalent to the human serum factor responsible for the complement-mediated lysis of MLVs and other retroviruses [158,159]. LVIF is produced by some but not all mouse strains, and genetic crosses between these strains show that LVIF is controlled by a single locus that maps to distal Chr 10 [160]. The gene responsible for this factor has not been identified.

### ERVs that interfere with exogenous infection

The mouse genome contains several resistance genes associated with production of MLV Env glycoproteins that are thought to restrict virus through receptor interference. These genes include *Fv4*, which blocks E-MLVs [161], and the genes *Rmcf *and *Rmcf2 *which restrict X/P-MLVs and, in the case of *Rmcf*, inhibit MCF MLV-induced disease [124,162-164]. Specific ERVs have been mapped to these resistance genes all of which are defective for virus production but have intact *env *genes: *Fv4 *and *Rmcf *have major deletions [161,165], and *Rmcf2 *has a stop codon that prematurely terminates integrase [124]. *Fv4*, *Rcmf*, and *Rcmf2 *reduce or downregulate activity of their cognate receptors, and *Fv4 *additionally has a defect in the fusion peptide of the TM*env*, so incorporation of this Env into virions in virus infected cells results in their reduced infectivity [166].

There is evidence of additional receptor blocking genes in *M. castaneus*. Three breeding lines of this species show similar restriction of P-MLV infection. Two of these lines, CAST/Rp and CAST/EiJ, carry *Rmcf2*. Backcross mice of the third line, CAST/Ncr, show evidence of two unlinked dominant resistance genes (#resistant/total = 87/123 = 0.71, χ^2 ^= 1.2, p = 0.3), and neither of these genes maps to Chr 5 (*Rmcf*) or Chr 18 (*Rmcf2*) (unpublished data). These loci, together with *Fv4 *and *Rmcf2*, may therefore be representative of a larger set of interference genes found in virus infected mouse species. That such co-opted Env genes are effective as host antiviral factors is confirmed by the identification of comparable interfering *env *genes in chickens, sheep and cats [167-169].

### *Fv1*

*Fv1 *is the oldest known retrovirus resistance gene [170] and represents a co-opted ERV sequence related to the *gag *gene of MuERV-L, a Class III ERV that is active in mice, but has no infectious virus counterparts [171,172]. The *Fv1 *sequence is found only in mice, and was acquired shortly after the origin of the *Mus *genus [173]. The laboratory mouse *Fv1 *has three well-characterized restriction alleles, and there are additional *Fv1-*like restrictions found in inbred strains and wild mouse species [173-176]. The three major laboratory mouse alleles, termed *Fv1^n^*, *Fv1^b^*, and *Fv1^nr ^*produce characteristic patterns of resistance to N-, B-, and NR-tropic MLVs. Cells with the *Fv1 *null allele are nonrestrictive [94,175], and NB-tropic viruses are not restricted by *Fv1*. *Fv1 *targets the virus capsid; the major determinant that distinguishes N- and B-tropic viruses is at CA position 110, but other target residues in this CA region have also been identified [176-180].

Because X-MLVs fail to infect cells of many laboratory mice, early studies used pseudotypes and recombinant viruses to suggest that X-MLVs are subject to *Fv1 *restriction [181,182]. Groom and colleagues [183] have more recently demonstrated that XMRV is unusual in that it is restricted in cells expressing either *Fv1^n ^or Fv1^b^*. Infection of X-MLV susceptible inbred strains suggests that XMRV is somewhat more sensitive to *Fv1^n^*, and also indicates that various mouse X-MLVs can be restricted by *Fv1 *[52]. XMRV carries the *Fv1^n ^*restricted residue at capsid target site 110, but its sensitivity to both *Fv1^n ^*and *Fv1^b ^*and the presence of additional substitutions in its CA gene suggest that the *Fv1 *target is more complex than previously appreciated.

### *Apobec3*

APOBEC3, like *Fv1*, is responsible for post-entry restriction of retroviral infection [184,185]. The mouse and human APOBEC3 genes are under strong positive selection suggesting an antiviral role in evolution [186,187]. There are 7 human APOBEC3 genes with differing antiviral activity against HIV-1 as well as MLVs; the single mouse APOBEC3 gene blocks HIV-1 and various mouse retroviruses [188-190]. mAPOBEC3 can restrict E-MLVs, and this gene maps to the site of the Friend virus restriction factor *Rfv3 *[191,192], a gene that influences the duration of viremia in virus-infected mice [193]. *Pmvs *but not *Xmv*s in the sequenced mouse genome show mutational patterns consistent with mAPOBEC3 silencing at the time of integration [25]. The sensitivity of infectious X/P-MLVs to APOBEC3 has not been determined, but XMRV is sensitive to inhibition by A3G, but is less sensitive or insensitive to A3A, A3B, A3C, A3F and A3H. XMRV is also more sensitive to mAPOBEC3 than is Moloney E-MLV [183,194].

The 2 laboratory mouse alleles of mAPOBEC3 vary in their restriction of Friend E-MLV replication and virus-induced disease, and differ in protein sequence, splicing pattern and expression levels [187,191,192,195]. The more antiviral allele of C57BL mice shows significantly higher levels of expression in mouse tissues [191,195], and this allele contains an *Xmv *LTR inserted into an intron [187]. This insertion introduces an intact LTR transcriptional enhancer, and all species and strains with this LTR show elevated mAPOBEC3 expression. This *Xmv*, acquired in virus-infected mice [187], may thus represent another example of an ERV sequence that is co-opted by the virus-infected host for an antiviral function, like *Fv1 *and *Rmcf*.

### Tetherin/BST2/CD317

Tetherin, also termed BST2, CD317 or HM1.24, is an interferon-inducible host factor that blocks the release of enveloped viruses by tethering budding particles to the cell surface [196]. Tetherin can be antagonized by the VPU accessory protein of HIV-1 or by the Nef protein of SIV or by the envelope glycoproteins of various immunodeficiency viruses [197-201]. The effectiveness of these viral protein antagonists is species specific [202], and these viral antagonists target different sites in tetherin [198]. Mouse E-MLV is subject to restriction by tetherin [196], and while the sensitivities of mouse X/P-MLVs to this host factor are unknown, XMRV has now been shown to be sensitive to human, monkey and mouse tetherins [183]. XMRV, as a simple gammaretrovirus, lacks the accessory proteins of the immunodeficiency viruses that antagonize tetherin action, and its Env glycoprotein does not interfere with tetherin activity [183].

## Conclusions

Multiple examples of xenotropism exist among the retroviruses. In addition to X-MLV, viruses that fail to infect cells of their apparent "home" species include the cat RD114-related viruses [203,204], GALV restriction in mice [205,206], and restrictions of avian leukosis viruses [207]. However, a half century of work on the MLVs and the availability of multiple inbred strains and wild mouse species have provided a unique look at the natural history of this particular virus-host relationship. The picture that emerges indicates that these X/P-MLVs were acquired as endogenous elements by *Mus *species with permissive receptors, and subsequent co-evolutionary modifications produced, among other adaptive phenotypes, "xenotropic" MLVs. The receptor mutations responsible for resistance to these X-MLVs were only recently acquired, and these restrictive receptors are only found among the inbred strains descended from early 20^th ^century fancy mouse colonies. It is now clear that the term "xenotropic" is somewhat of a misnomer for mouse viruses that actually infect cells of all *Mus *species and many common strains of laboratory mice, and that infect more non-*Mus *mammals than the so-called broad host range polytropic MLVs.

The interacting interfaces of host and pathogen are co-evolutionary battlegrounds, and the effects of the ratchet-like mutational process affecting these entities are particularly obvious for restriction factors like *Xpr1 *and *Fv1*. The battleground at the cell surface has produced at least 6 host range X/P-MLV variants that interact with different but overlapping sets of determinants on the XPR1 receptor. At the same time, other factors likely contribute to diversifying selection on the X/P-MLV viral Env glycoprotein, such as the *Rmcf-*type interfering ERVs and the inactivating LVIF serum factor. The resulting Envs not only vary in tropism, but these viruses have also evolved alternative mechanisms of transmission. Although germline PMV ERVs are effectively silenced, and the Env glycoproteins of infectious recombinant P-MLVs have narrow receptor requirements and reduced receptor binding efficiency, these multiple mechanisms ensure their transmission and also contribute to pathogenicity.

MLV ERVs, as part of the host genome, are also shaped by evolutionary processes. ERV insertions introduce novel regulatory and protein coding sequences into the host genome. While most are silenced, some are co-opted for cellular functions, and the most easily recognized of these domesticated ERVs are those that are linked to antiviral functions. Such ERVs include the oldest recognized restriction factor, *Fv1*, [170] as well as X/P-MLV ERV insertions that include the multiple *Rmcf*-like interference genes, and regulatory elements like the mAPOBEC associated *Xmv *LTR.

The worldwide distribution of mice that carry MLVs and the broad host range of the X-MLVs suggest that we are only beginning to describe what may be common and widespread interspecies transmissions. The phenotypic diversity among the MLVs doubtless influences the likelihood of their transspecies transmission, and the invading viruses are then subject to additional co-evolutionary pressures. We do not know the evolutionary path taken by XMRV to humans, but multiple sequence and functional variations distinguish this virus from its MLV progenitors. The consequences of this transspecies MLV invasion are unclear, although XMRV pathophysiology is now being evaluated in other species, including primates [208] as well as in *Mus *species, like *M. pahari*, that are permissive for X-MLV and XMRV infection [209]. The fact that all mice carrying infectious X-MLVs have one of 3 restrictive receptors suggests that unchecked X-MLV infection is likely to be deleterious, and the recognition that many mice carry permissive receptors now makes it possible to describe the pathogenic consequences of exogenous X-MLV infection in their natural host. Further characterization of these viruses should further elucidate their evolutionary past and describe their pathogenic potential and the adaptations that favor co-existence of these infectious agents and their new human hosts.

## Competing interests

The author declares that she has no competing interests.
